# Leisure constraints and the negotiation of structural relationships: a case study of scuba diving enthusiasts

**DOI:** 10.3389/fspor.2025.1586601

**Published:** 2025-06-16

**Authors:** Jing Chen, Zihan Yu, Ruiyang Ni

**Affiliations:** ^1^ School of Marxism, Zhejiang Shuren University, Hangzhou, Zhejiang, China; ^2^ Hangzhou International Urbanology Research Center & Zhejiang Urban Governance Studies Center, Hangzhou, Zhejiang, China; ^3^ Zhejiang Leisure Association, Hangzhou, Zhejiang, China; ^4^ School of Public Affairs, Zhejiang Shuren University, Hangzhou, Zhejiang, China; ^5^ School of Humanities & Foreign Languages, Zhejiang Shuren University, Hangzhou, Zhejiang, China

**Keywords:** SCUBA diving, leisure constraints, constraint negotiation, cognitive strategies, behavioral strategies

## Abstract

**Background:**

Scuba diving has emerged as a popular recreational activity in China over the past two decades, yet academic research on this sport from the perspective of leisure studies remains limited. This study explores the relationship between leisure constraints and constraint negotiation among scuba diving enthusiasts, aiming to fill this research gap.

**Method:**

This study employed a mixed-methods approach, combining in- depth interviews with 20 scuba diving enthusiasts and the Positive and Negative Affect Schedule (PANAS) for survey and analysis. The interviews focused on the participants' leisure motivations, the constraints encountered at different stages of their diving careers, and the negotiation strategies they employed.

**Results:**

The findings revealed that scuba diving enthusiasts tend to use cognitive negotiation strategies when addressing personal and interpersonal constraints, while predominantly employing behavioral negotiation strategies when dealing with structural constraints. A structural relationship was identified between leisure constraints and constraint negotiation, indicating that the type of constraint influences the negotiation strategy employed.

**Conclusions:**

This study provides empirical support for the structural relationship between leisure constraints and constraint negotiation, enriching the materials available for leisure research. Future research is recommended to expand the sample size and further explore the underlying mechanisms of this relationship, as well as to consider the authenticity and accuracy of respondents' self-reports.

## 1 Introduction

### 1.1 Background

Scuba diving is a highly popular recreational activity in Western Europe and North America, regions with a strong foundation in marine sports. According to statistics, in the United States alone, 40 million individuals partake in diving activities worldwide annually. Among the 200 million Americans, 60 million hold scuba diving certifications, representing 30% of the population. In France, with a population of only 40 million, there were already 2,000 diving clubs by the end of the 20th century. To date, formal diving clubs in Mainland China have only been established for a little over two decades. Despite the relatively late onset of recreational diving in China, the activity has experienced remarkable growth, with a rapid expansion in the number of participants (1). This trend demonstrates the immense potential and broad market demand for this sport. However, despite its global popularity, academic research on scuba diving from the perspective of leisure studies remains remarkably limited.

This paper first introduces the nature of scuba diving as a recreational activity. It then examines scuba diving through the lens of leisure constraints and constraint negotiation. Finally, through interviews with 20 amateur scuba diving enthusiasts, primary data are obtained and analyzed to derive a model of the structural relationship between leisure constraints and constraint negotiation.

### 1.2 Recreational diving evolution

Covering 70% of the Earth's surface, the ocean has long been a source of immense fascination and endless mystery for humanity. Since ancient times, driven by curiosity and a spirit of fearlessness, humans have continuously explored the unknown depths of the ocean. The most direct form of contact with the ocean is through diving. As early as 2,800 years ago, during the zenith of Mesopotamian culture, records indicate that diving activities were conducted for military purposes (2). In modern times, diving initially emerged as an activity undertaken by experienced professional divers to perform underwater surveys, salvage operations, repairs, and underwater engineering tasks. This type of diving, requiring specialized skills and training, is referred to as commercial diving or professional diving (3).

Subsequently, diving evolved to serve new purposes, transforming into a recreational activity focused on underwater sightseeing, with the goals of physical exercise, mental relaxation, and leisure enjoyment. This form of diving is known as recreational diving (4).

In recent years, advancements in diving equipment have propelled the rapid development of recreational diving. Consequently, the number of individuals engaging in and expressing interest in diving has increased significantly. In advanced countries such as Europe and the United States, recreational diving gained popularity as early as the 1950s. The development of recreational diving in China, however, is relatively recent (5). Prior to 1995, this field was virtually non-existent in China. The establishment of the China International Diving Club on March 18, 1995, marked the beginning of a rapid development phase for recreational diving in China. Over the past two decades, recreational diving in China has developed structured club organizations and stable participant groups, with studies in Hong Kong highlighting the role of clubs in promoting organized diving practices and behavioral norms (6).

Within the realm of recreational diving, there are three primary types of activities: snorkeling, free diving, and scuba diving. Among these, scuba diving refers to diving activities in which divers carry their own underwater breathing apparatus. In this study, the examination of recreational diving activities specifically focuses on scuba diving. All selected interviewees for this survey are divers participating in scuba diving activities.

### 1.3 Existing studies on diving activities

As a recreational sport, scuba diving initially emerged in relatively developed regions such as Western Europe and North America, and research on this activity also originated from these areas. Initially, the majority of researchers examined the environmental impacts of diving activities from an ecological perspective. For example, a nine-year study titled Sustainability of Scuba Diving Tourism on Coral Reefs of Saba was completed by Julie P. Hawkins, Callum M. Roberts, David Kooistra, Ken Buchan, and Susan White (7). Another study, Moderator and Mediator Effects of Scuba Diving Specialization on Marine-Based Environmental Knowledge-Behavior Contingency (2005), was conducted by Brijesh Thapa, Alan R. Graefe, and Louisa A. Meyer (8) through a survey of 370 scuba divers.

In addition to ecological perspectives, some scholars have examined recreational diving from a tourism standpoint. Ghazali Musa (9) published Sipadan: A SCUBA-diving paradise: An analysis of tourism impact, diver satisfaction and tourism management. Martin McCarthy (10) and colleagues conducted research on consumer satisfaction titled Customer Satisfaction and Scuba-diving: Some insights from the deep. Review studies in this area include Scuba Diving Tourism: Introduction to Special Issue by Ghazali Musa and Kay Dimmock (11), as well as Scuba Diving Tourism by Michael Lück (12).

Psychological perspectives have also been explored in the study of scuba diving. Kay Dimmock (13) published Finding Comfort in Adventure: Experiences of Recreational SCUBA Divers. Tah Fatt Ong (14) and colleagues examined the underwater behavior of recreational divers through attitude-behavior theories in their study titled An Examination of Recreational Divers' Underwater Behavior by Attitude-Behavior Theories. Stephanie Merchant (15) explored the sensory and bodily experiences of scuba diving in her work titled Negotiating Underwater Space: The Sensorium, the Body and the Practice of Scuba-diving.

In comparison, international researchers have conducted more specialized and segmented studies on scuba diving. In China, however, the popularity of recreational diving is still relatively low, resulting in limited domestic research. Studies from the perspective of leisure studies are particularly scarce. Initially, domestic research on diving in China primarily focused on the psychological and physiological aspects of professional divers. For example, a personality psychology survey of 189 diving trainees was conducted by Yu Qinghua, Yan Guohua, and Zhao Kunming (16). Xiao Chenghua, Li Shide, and Bai Lishan (17) investigated the physical and mental health of 70 divers. A psychological function test method for divers was proposed by Jing Yanlin and Gao Guizhen (18). Wang Jiali, Miao Luqing, Jiang Zhenglin, and Dai Jiajun (19) jointly developed a preliminary scale for assessing the mental health dimensions of professional divers.

The second category of relatively more abundant literature consists of reviews on the history and current state of recreational diving in China. For instance, Su Xiong (20) published a study on recreational diving. Zhang Qiang, Chang Qing, and Yuan Hancheng (21) explored the historical significance of the initial stage of modern diving sports in China. Yuan Feng (22) conducted a bibliometric analysis of recreational diving research literature in China over the past decade.

### 1.4 Leisure constraints and negotiation

Leisure constraints refer to the factors that restrict or hinder the quality, duration, intensity, frequency, and other aspects of participation in leisure activities, thereby impeding the enjoyment of leisure (23).

Thus, the purpose of our research on leisure constraints is to “investigate the factors that limit the formation of leisure preferences or prevent people from participating in and enjoying leisure activities” (24). Typically, research on the negative factors that determine individual leisure choices—namely, leisure constraints—can help us explain the relationships between individuals' leisure attitudes, preferences, and ultimate leisure behaviors, and may even provide new insights into conclusions previously based on researchers' subjective perceptions.

Early theories of leisure constraints posited that constraints were static, meaning that leisure constraints were almost insurmountable limitations or obstacles to participation in leisure activities, i.e., with constraints—no participation, without constraints—participation. This paradigm and model led researchers to overlook the impact of leisure constraints on participants and the other outcomes beyond non-participation that these constraints might bring.

In the 1980s and 1990s, skepticism toward early leisure constraint research led to significant developments in constraint theory. First, the term “leisure barriers” was replaced by “leisure constraints”. Subsequently, Crawford and Godbey introduced the most important concepts and models in leisure constraint theory research. In their 1987 article “Reconceptualizing Barriers to Family Leisure” published in Leisure Sciences, they made two major contributions: First, Crawford and Godbey (25) argued that constraints not only affect participation and non-participation but also influence preferences. In other words, a lack of interest in or awareness of an activity is also subject to or can be explained by constraints. Second, they expanded the range of factors that might influence behavior. That is to say, in addition to structural constraints, leisure constraints also include intrapersonal and interpersonal factors.While Crawford & Godbey's three-dimensional model of leisure constraints (intrapersonal, interpersonal, and structural) provides a useful theoretical framework, its application in the Chinese context requires careful consideration of cultural, economic, and social specificities. For instance, family dynamics in China often play a significant role in shaping individual leisure choices, while economic pressures and limited access to resources further distinguish the Chinese context from Western settings (26).The latter two, compared to the secondary structural factors, are more direct and more likely to influence leisure choices because they directly affect the formation of leisure preferences as individual internal factors and social factors, while structural factors influence participation before the actual engagement, after the formation of leisure preferences.

Since then, research on leisure constraints has continued to evolve. Scott (27) first introduced the concept of “negotiation” into leisure constraint theory in 1991. He pointed out that people often adopt a “negotiation” attitude towards leisure constraints, meaning that the presence or absence of constraints does not determine whether people participate in leisure. The decisive factor is the outcome of negotiation with the constraints, which usually involves adjustment rather than direct cancellation of participation. Shaw, Brawner, and Mcaleer (28) found that individuals with higher constraints participate in leisure activities more frequently than those with lower constraints, which is contrary to the intuitive view in the constraint-participation model.

**Figure 1:**
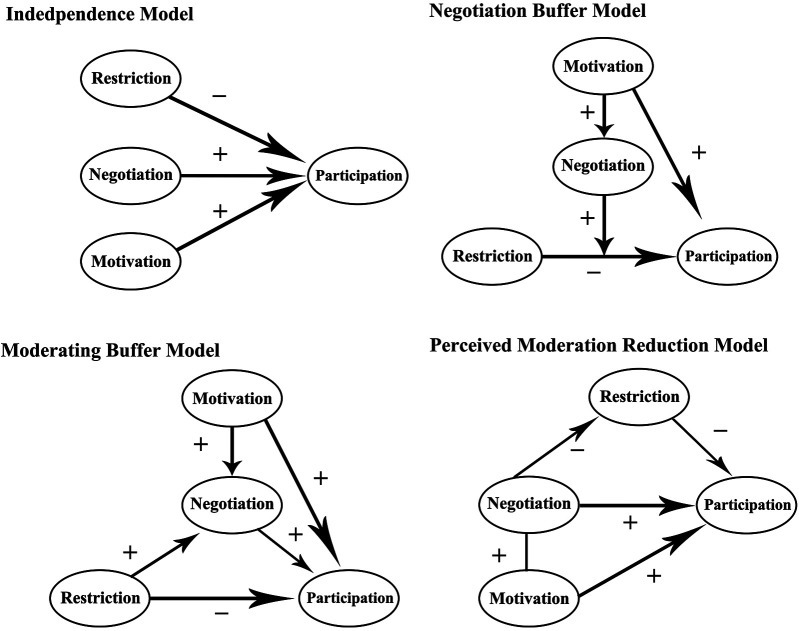
The competitive model of constraint negotiation process (30).

Initially, constraint negotiation strategies were divided by Jackson (29) into two major categories: cognitive strategies (adjusting expectations, changing interests, etc.) and behavioral strategies (time management, financial management, skill learning, improving interpersonal relationships, etc.). By the early 21st century, Huber and Mannell established a four-factor model in the negotiation process from the perspective of the relationships among motivation, constraints, negotiation, and participation (see Figure 1), further refining the constraint-negotiation theory. The introduction of concepts from social cognitive psychology, such as self-efficacy, selective optimization with compensation theory, self-construction, and social identity, into the constraint-negotiation theory has been even more helpful in explaining the internal mechanisms of this process.

**Figure 2:**
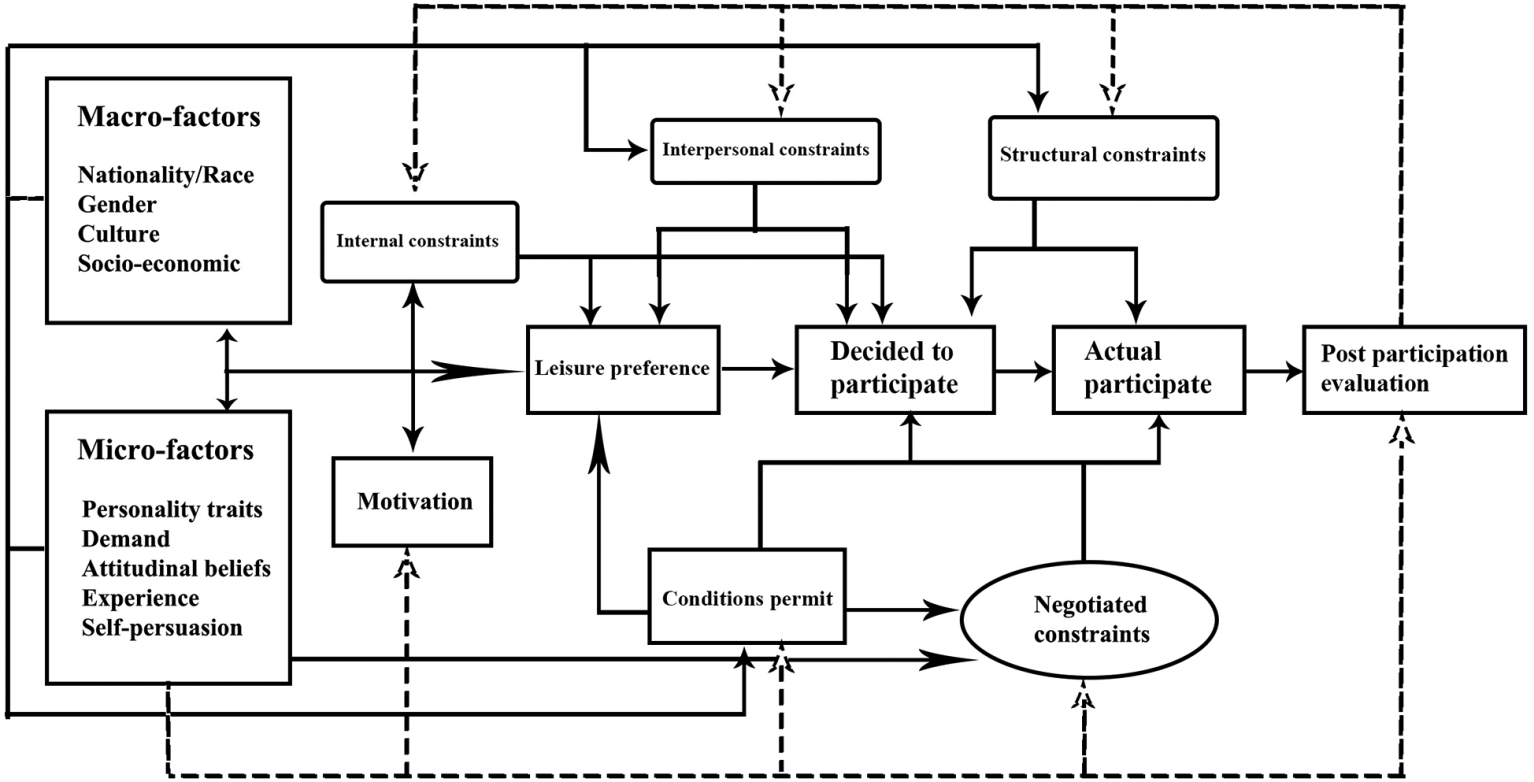
The outdoor recreation leisure constraints model (24).

Subsequently, Walker and Virden (31), building upon the theories and models of numerous scholars, proposed a relatively comprehensive revised model of leisure constraints for outdoor recreation. Figure 2 integrates the entire process of leisure activities, incorporating mechanisms of preference, decision-making, constraints, negotiation, behavior, as well as the influence of macro- and micro-level factors. The significance of leisure constraint research in the field of leisure studies over the past two decades is profound. This is because research on leisure constraints is not merely concerned with whether individuals participate in certain leisure activities; rather, it involves multiple aspects, including leisure motivation, preference, and experience. Correspondingly, it holds implications for leisure services and the planning of leisure spaces.

## 2 Materials and method

The study examines the leisure constraints and negotiation strategies among scuba diving enthusiasts, analyzing the structural relationships between them through the collection and analysis of primary data. The research employs in-depth interviews as the primary method.

### 2.1 Interview sample description

A total of 20 divers participated in the interviews, comprising 10 females and 10 males, with ages ranging from 25 to 52 years. All interviewees were non-professional divers, with diving experience varying from one to five years. Their occupations, income levels, and places of residence were diverse, although the majority were single and had high levels of education. The sample selection was representative. Detailed analysis is presented in Table 1.

**Table 1:** Basic information of respondents.

Category	Gender	Age	Job	Working age	Marital status	Educational level	Monthly income (yuan)	Residence
F1	Female	30	Company staff	2 years	Married	Bachelor degree	Twenty thousand	Beijing
F2	Female	30	Online store owner	5 years	Unmarried	Bachelor degree	Thirty thousand	Dalian
F3	Female	28	Clerk	3 years	Unmarried	Bachelor degree	Ten thousand	Nanjing
F4	Female	37	Civil servant	4 years	Unmarried	Master's degree	More than Ten thousand	Wuhan
F5	Female	35	Hospital administrator	3 years	Unmarried	Bachelor degree	Eight thousand	Guiyang
F6	Female	40	Owner operator	5 years	Unmarried	Junior college diploma	Twenty thousand	Nanjing
F7	Female	29	Company staff	1 year	Unmarried	Junior college diploma	Twenty thousand	Hong Kong
F8	Female	32	Architect	2 years	Unmarried	Bachelor degree	Six thousand	Shenyang
F9	Female	25	Corporate treasurer	1 year	Unmarried	Bachelor degree	Five thousand	Dongguan
F10	Female	40	Tourism management personnel	1 year	Married	Bachelor degree	Forty thousand	Guangzhou
M1	Male	35	Employees of state-owned enterprises	3 years	Unmarried	Bachelor degree	Five thousand	Xi’an
M2	Male	30	Marketing manager	1 year	Unmarried	Bachelor degree	Fifteen thousand	Shanghai
M3	Male	39	Civil servant	5 years	Unmarried	Bachelor degree	Six thousand	Hangzhou
M4	Male	28	Foreign trade salesman	2 years	Unmarried	Bachelor degree	Forty thousand	Foshan
M5	Male	42	Engineer	5 years	Unmarried	Junior college diploma	More than fifty thousand	Shanghai
M6	Male	28	Graphic designer	2 years	Unmarried	Bachelor degree	Twelve thousand	Hangzhou
M7	Male	33	Aircraft commander	2 years	Married	Bachelor degree	More than thirty thousand	Chongqing
M8	Male	40	Company manager	2 years	Married	Bachelor degree	Twenty to thirty thousand	Guangzhou
M9	Male	52	Freelancer	3 years	Married	Junior college diploma	Ten thousand	Shanghai
M10	Male	35	Reporter	3 years	Unmarried	Bachelor degree	More than Ten thousand	Guangzhou

### 2.2 Interview protocol

The interview focuses on the respondents' leisure motivations and preferences, the different leisure constraints encountered and negotiation strategies employed at the beginning, advancement, and peak stages of their recreational diving careers, as well as their levels of leisure participation and satisfaction. The specific questions are as follows:

(1) When did you start diving, and what motivated you to begin? (Motivation, formation of leisure preference)
(2) What barriers did you encounter when you first started recreational diving, and what were your achievements? What prompted you to take the certification exam for recreational diving? (Leisure constraints and negotiation strategies at the career onset)
(3) After obtaining the Open Water certification, why did you continue diving and pursue higher-level certifications? (Leisure constraints and negotiation strategies during career advancement)
(4) What is your current diving certification level? (e.g., AOW, Rescue Diver, Master Scuba Diver, Dive Master) How long did it take to achieve this level? What were the greatest gains and obstacles during this process? At which stage did diving bring you the most satisfaction? (Leisure constraints, negotiation strategies, leisure satisfaction, and participation)
(5) Have you considered further advancing your diving skills? What are the reasons for or against continuing to advance? (Leisure constraints and negotiation strategies at the career peak)

### 2.3 Positive and negative affect schedule (PANAS)

The study examines the subjective expectations prior to participation and the objective feelings following participation in diving activities among interviewees, focusing on intrapersonal and interpersonal constraints within the context of leisure constraints. The scale consists of 12 items, utilizing a 5-point Likert scale for scoring, where 1 indicates the weakest level of expectation or actual feeling, and 5 indicates the strongest.All statistical analyses for the PANAS scale, including reliability (Cronbach's alpha) and validity (factor analysis) assessments, were performed using the Statistical Package for the Social Sciences (SPSS), version 29.0 (IBM Corp., Armonk, NY, USA).

(1) Feeling happy and excited before participating in diving activities.
(2) Feeling nervous and anxious before participating in diving activities.
(3) Expecting to relieve stress and relax through diving activities.
(4) Expecting to feel more energetic and motivated through diving activities.
(5) Feeling proud to be a diver (due to skill improvement).
(6) Feeling pressured to be a diver (due to skill inadequacy or lack of support from friends and family).
(7) Feeling more inspired after participating in diving activities compared to before.
(8) Feeling guilty after participating in diving activities.
(9) Feeling more determined to continue engaging in diving activities after participation.
(10) Feeling timid or even depressed after participating in diving activities (due to perceived incompetence).
(11) Losing interest in continuing diving activities (due to fatigue, skill inadequacy, or lack of companions).
(12) Looking forward to continuing diving activities (expecting to make more friends).

To ensure the reliability of the PANAS scale used in this study, a Cronbach's alpha analysis was conducted. The results are presented in Table 2. The overall Cronbach's alpha coefficient for the scale was 0.769, indicating good internal consistency. Individual items showed varying levels of contribution to the overall reliability, with corrected item-total correlations (CITC) ranging from −0.226 to 0.736. The alpha coefficients if items were deleted ranged from 0.728 to 0.823, further supporting the scale's reliability.

**Table 2:** Cronbach's alpha analysis for PANAS scale.

Item	Corrected item-total correlation (CITC)	Alpha if item deleted	Cronbach's α
Feeling happy and excited before participating in diving activities	0.580	0.754	0.782
Feeling nervous and anxious before participating in diving activities.	0.182	0.791	
Expecting to relieve stress and relax through diving activities	0.488	0.760	
Expecting to feel more energetic and motivated through diving activities	0.667	0.738	
Feeling proud to be a diver (due to skill improvement)	0.736	0.728	
Feeling pressured to be a diver (due to skill inadequacy or lack of support from friends and family)	0.360	0.773	
Feeling more inspired after participating in diving activities compared to before	0.671	0.736	
Feeling guilty after participating in diving activities	0.214	0.783	
Feeling more determined to continue engaging in diving activities after participation	0.511	0.757	
Feeling timid or even depressed after participating in diving activities (due to perceived incompetence)	0.303	0.779	
Losing interest in continuing diving activities (due to fatigue, skill inadequacy, or lack of companions)	−0.226	0.823	
Looking forward to continuing diving activities (expecting to make more friends)	0.554	0.751	

The standardized Cronbach's alpha coefficient was 0.769.

To assess the validity of the PANAS scale, a factor analysis was conducted. The results are presented in Table 3. The factor loadings, communalities, and other relevant statistics are shown below. The Kaiser-Meyer-Olkin (KMO) value was 0.796, indicating sampling adequacy. Bartlett's test of sphericity was significant (*χ*^2^ = 364.246, df = 66, *p* = 0.000), supporting the factorability of the correlation matrix. Three factors were extracted, explaining a cumulative variance of 69.741% after rotation. The factor loadings indicated a clear factor structure, supporting the construct validity of the PANAS scale.

**Table 3:** Factor analysis results for PANAS scale.

Item	Factor loading			Communality
	Factor 1	Factor 2	Factor 3	
Feeling happy and excited before participating in diving activities	0.774	−0.177	0.344	0.749
Feeling nervous and anxious before participating in diving activities.	−0.065	0.791	0.033	0.631
Expecting to relieve stress and relax through diving activities	0.744	−0.227	0.280	0.684
Expecting to feel more energetic and motivated through diving activities	0.884	0.082	−0.155	0.812
Feeling proud to be a diver (due to skill improvement)	0.834	0.215	−0.003	0.741
Feeling pressured to be a diver (due to skill inadequacy or lack of support from friends and family)	0.121	0.791	0.155	0.664
Feeling more inspired after participating in diving activities compared to before	0.814	0.175	−0.065	0.697
Feeling guilty after participating in diving activities	0.043	0.229	0.847	0.771
Feeling more determined to continue engaging in diving activities after participation	0.811	−0.150	−0.039	0.681
Feeling timid or even depressed after participating in diving activities (due to perceived incompetence)	0.047	0.799	0.085	0.648
Losing interest in continuing diving activities (due to fatigue, skill inadequacy, or lack of companions)	−0.524	0.471	0.455	0.704
Looking forward to continuing diving activities (expecting to make more friends)	0.761	0.066	−0.059	0.586
Eigenvalue (Before Rotation)	4.833	2.521	1.015	–
Variance Explained (%) (Before Rotation)	40.276%	21.008%	8.457%	–
Cumulative Variance Explained (%) (Before Rotation)	40.276%	61.284%	69.741%	–
Eigenvalue (After Rotation)	4.825	2.358	1.186	–
Variance Explained (%) (After Rotation)	40.211%	19.646%	9.884%	–
Cumulative Variance Explained (%) (After Rotation)	40.211%	59.857%	69.741%	–
Kaiser-Meyer-Olkin (KMO) Measure of Sampling Adequacy	0.796			–
Bartlett's Test of Sphericity: *χ*²	364.246			–
*df*	66			–
*p*	0.000			–

## 3 Results

During the interview process, participants predominantly discussed their experiences and perceptions of diving across several key dimensions: the initiation of their recreational diving careers, the constraints encountered during skill advancement and participation in diving activities, and the corresponding negotiation strategies. They also addressed the new social opportunities afforded by diving and the ways in which participation in diving activities enhanced their life satisfaction.

### 3.1 Diving enthusiasts: constraint negotiation to certification

Scuba diving, as a leisure activity, is distinct from other recreational pursuits. Notably, before becoming a certified scuba diver, individuals must undergo training and examination by a professional organization (32). Only after obtaining the relevant certification are they permitted to participate in organized scuba diving activities. Given the inherent risks and technical nature of this activity, specialized skills and knowledge are required. In the absence of such certification, individuals with an interest in diving may only engage in introductory diving experiences. For example, F3 mentioned, “*After my first introductory dive, I was completely hooked by the underwater world and couldn't wait to get certified”.* F2 also mentioned, “*At first, it was hard to find diving buddies, but after joining a diving club, I met many like-minded people, which made the whole experience more enjoyable”.* These experiences, lacking autonomy, professionalism, and technicality, are not classified as scuba diving activities in this study. The sample selected for this research consists exclusively of certified recreational divers who have obtained their scuba diving certification.

The Professional Association of Diving Instructors (PADI) is currently the most popular and largest global diving training organization. PADI has 25,000 instructors worldwide, issuing over 500,000 diving certifications annually. It also trains its own instructors and examiner instructors, and certifies dive shops and clubs where these instructors are based. All the instructors and divers known to the author, including the author's own certification, are issued by PADI.

#### 3.1.1 Constraints and coping strategies for scuba diving enthusiasts

All interviewees began their narratives with how they developed an interest in diving. The majority were travel enthusiasts with experiences in international travel, particularly favoring island tourism. Among them, many who enjoyed swimming or had participated in snorkeling activities were exposed to the underwater world's breathtaking and novel scenery through various media. Alternatively, they had directly experienced the grandeur and allure of coral reefs and fish shoals during snorkeling. These experiences motivated them to engage in scuba diving through introductory dives organized by diving clubs, allowing them to more closely experience the ocean's wonders. After one or two introductory dives, interviewees gained a basic understanding of the rules and skills of scuba diving. Having experienced the joy of the activity, they all chose to pursue certification as recreational divers.

In deciding to undertake PADI diver training, each individual encountered certain leisure constraints, predominantly structural factors such as time and financial limitations, as well as interpersonal constraints, including family concerns and the lack of companions. At this stage, intrapersonal constraints were less prevalent, as most interviewees had already participated in introductory dives and had developed a preference and desire for this leisure activity. However, a few interviewees mentioned mild intrapersonal psychological constraints, such as uncertainty about their ability to succeed in the activity.

In the Chinese context, personal constraints such as lack of confidence or fear of safety risks are often exacerbated by societal attitudes toward high-risk activities, which are less normalized compared to Western countries (33). F6 mentioned the concerns of her family about diving activities: “*My family is not overly worried about the expenses I spend on diving. I pay for diving myself. They aren't too worried about me in this regard. However, if my parents don't support me, there is not much I can do. They think that obtaining certification and diving are not only very risky and may be life-threatening, but also useless, so they don't support me”.*

During this phase, interviewees primarily employed behavioral strategies for constraint negotiation. Given their initial enthusiasm for diving, the negotiation approaches often involved promoting the activity to family or friends and joining clubs to find like-minded individuals, thereby improving social support.

#### 3.1.2 Constraints and strategies for advancing in scuba diving

In PADI diving courses, the advanced programs specifically designed for non-professional divers are divided into several stages: Open Water Diver, Advanced Open Water Diver (AOW, with a depth limit of 30 meters and qualifications for night diving, boat diving, and open-water cavern diving), Rescue Diver, and Master Scuba Diver (the highest honor for recreational divers). For those aspiring to become professional instructors, there are the Diver Master and Open Water Scuba Instructor courses. These advanced courses are also pursued by amateur diving enthusiasts to elevate their diving skills to a higher level (34).

During the advanced stages of diving participation, the leisure constraints encountered by respondents differ from those experienced at the onset of learning scuba diving. At this stage, intrapersonal constraints become more pronounced. M1, a state-owned enterprise employee, says: “*I've gone diving more often, but I still worry it's not good for my boss to know I often go out for fun. So, on my personal social media, I block my leaders and colleagues. On some level, this is also a constraint for me”.*As PADI courses progress in complexity, the stress associated with scuba diving activities becomes more evident, manifesting not only in time and financial pressures but also in significant psychological stress. For instance, enthusiasm for the activity may wane compared to the initial stages, with extensive training and examinations required for advancement diminishing the enjoyment of diving. In some cases, an unpleasant or unsafe diving experience may even lead to the consideration of discontinuing participation. Family opposition, a significant interpersonal constraint in China, often stems from cultural values emphasizing safety and stability, which may lead families to discourage participation in activities like scuba diving. F8 shared her view on family opposition: “*Family opposition often stems from safety concerns. For instance, when I plan to dive in faraway places, they think it's dangerous. But I usually explain to them that diving is actually a safe sport as long as I follow the rules”.*

In this phase, scuba diving enthusiasts primarily employ cognitive strategies such as enhancing self-efficacy and identity, alongside behavioral strategies focused on improving social support, particularly seeking assistance from peer groups. As F8 stated, “*When I faced difficulties in advanced courses, I kept telling myself that I've come this far and I can do it. Joining a peer group also helped me a lot as we shared experiences and supported each other”*. The limited availability of training resources was another challenge. M9 stated, *“In China, there are not many high-quality diving training resources. I had to travel to different cities several times to find suitable instructors, which was both time-consuming and costly*”. Structural constraints in China are further compounded by the limited availability of affordable training resources and the high cost of diving equipment, which restrict access to the activity for many enthusiasts (35). F1 mentioned in a humorous way: “*Another thing is the cost. For example, an 8-day trip to the Galapagos costs over 70,000 yuan, which is my whole year's diving budget. I think it's not worth it. I'd better go to other places more times. I can't afford such a price now. I plan to dive there when I'm 60. It's not something I can afford now. I can go, but after spending all that money, I still feel a bit”.*

### 3.2 Leisure constraints

**Figure 3:**
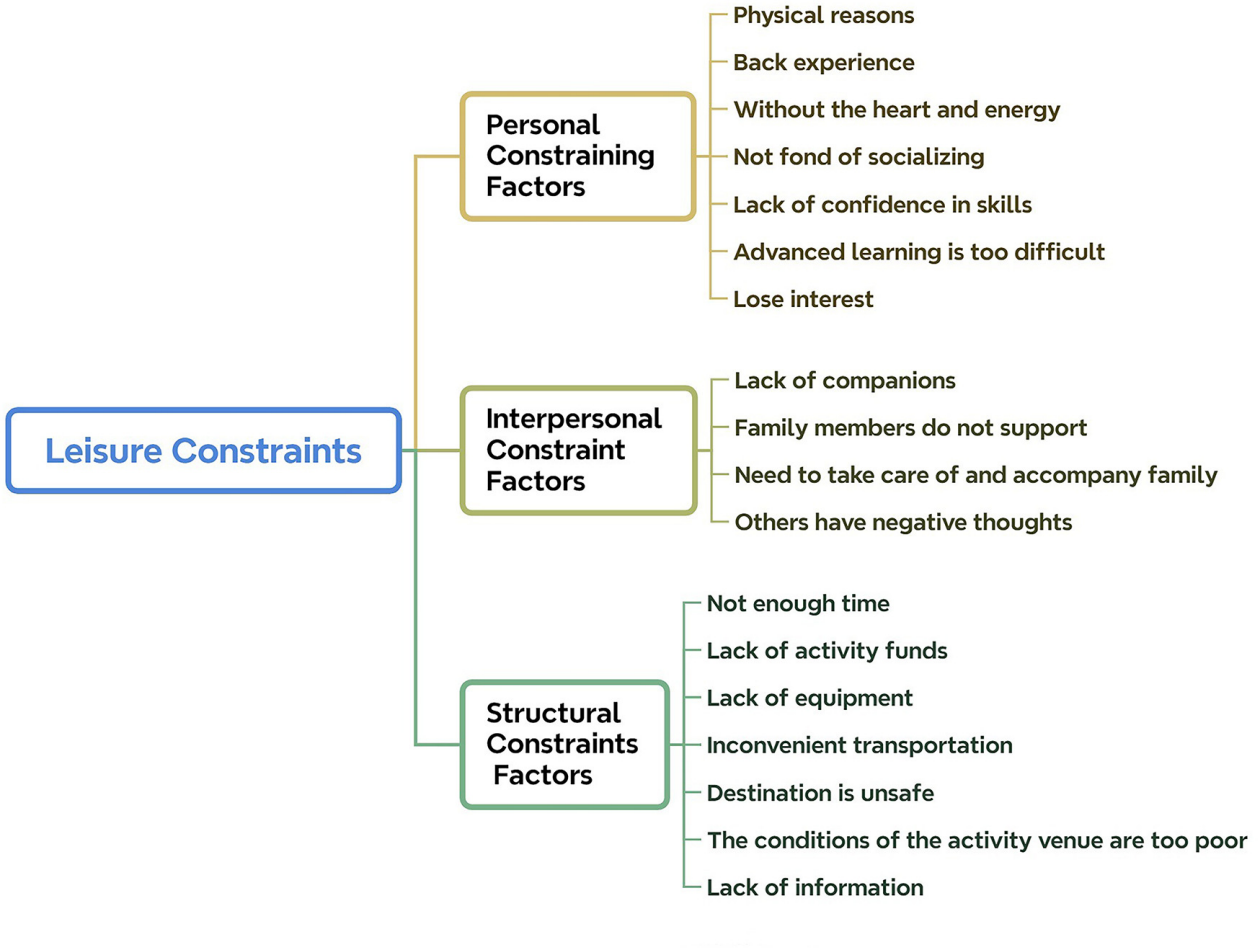
Leisure constraints model.

The leisure constraint model employed in this study is categorized according to the three dimensions proposed by Crawford and Godbey: intrapersonal constraints, interpersonal constraints, and structural constraints. Based on the interview results, these constraints are summarized into 18 factors. The specific classification is shown in Figure 3.

### 3.3 Constraining negotiation strategies

**Figure 4:**
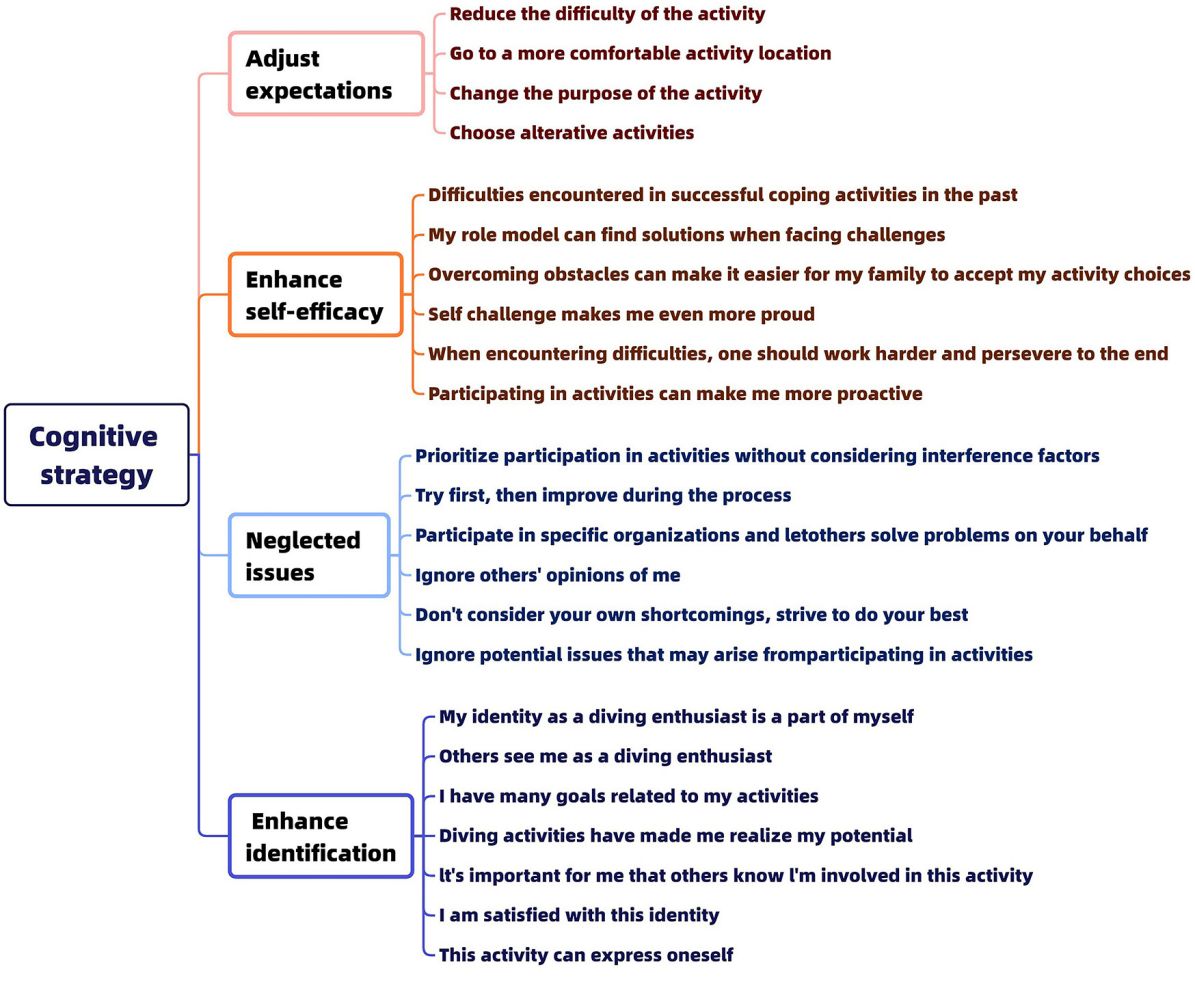
Cognitive strategies for constrained negotiation.

**Figure 5:**
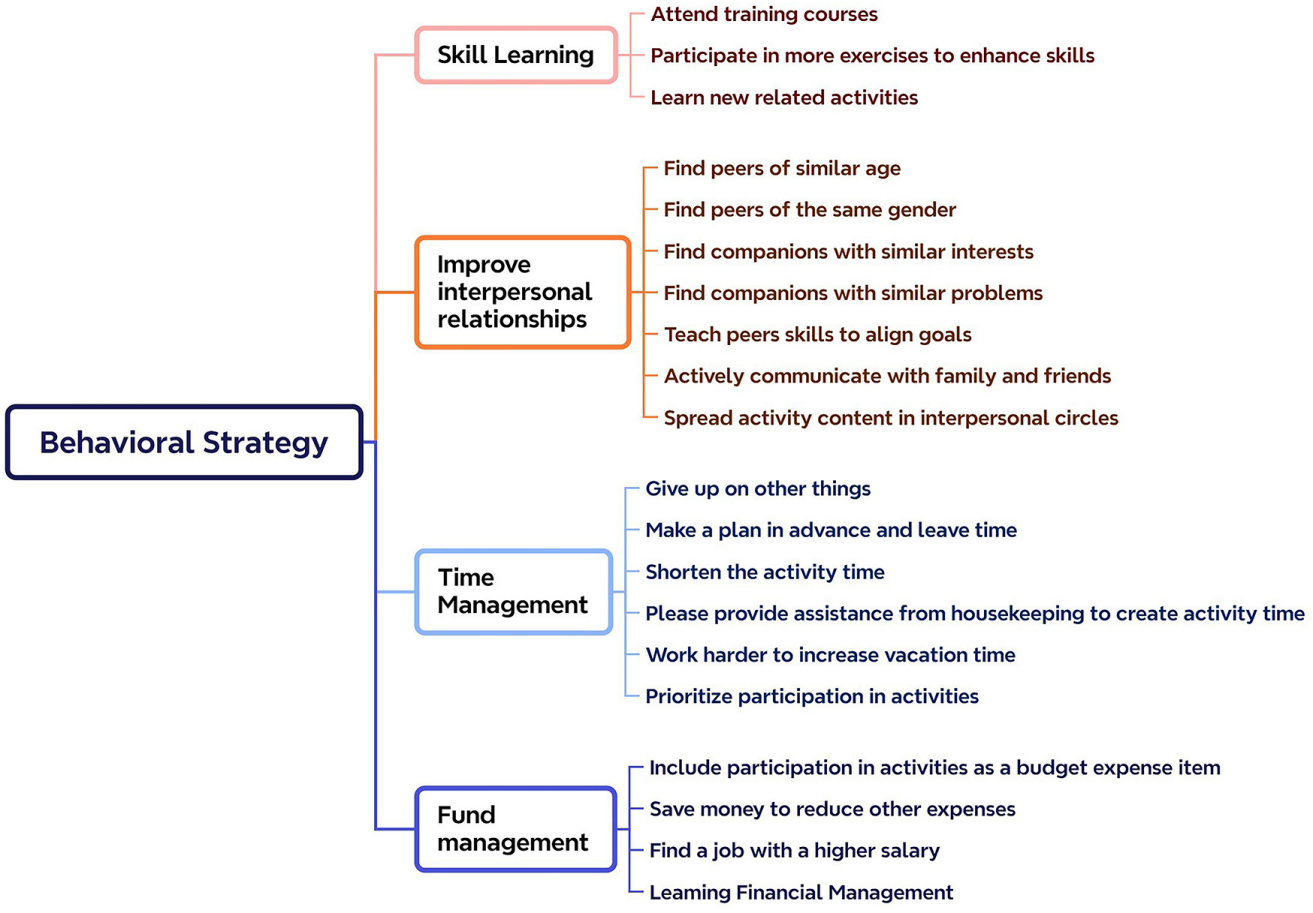
Strategies for constraining negotiation behavior.

The study's constraint negotiation strategy model is based on Jackson's cognitive and behavioral strategies, which, according to the interview data, may be summed up as 23 cognitive and 20 behavioral methods. The particular classification is displayed in Figures 4, 5.

### 3.4 Leisure constraints—consultative structural relationships

**Figure 6:**
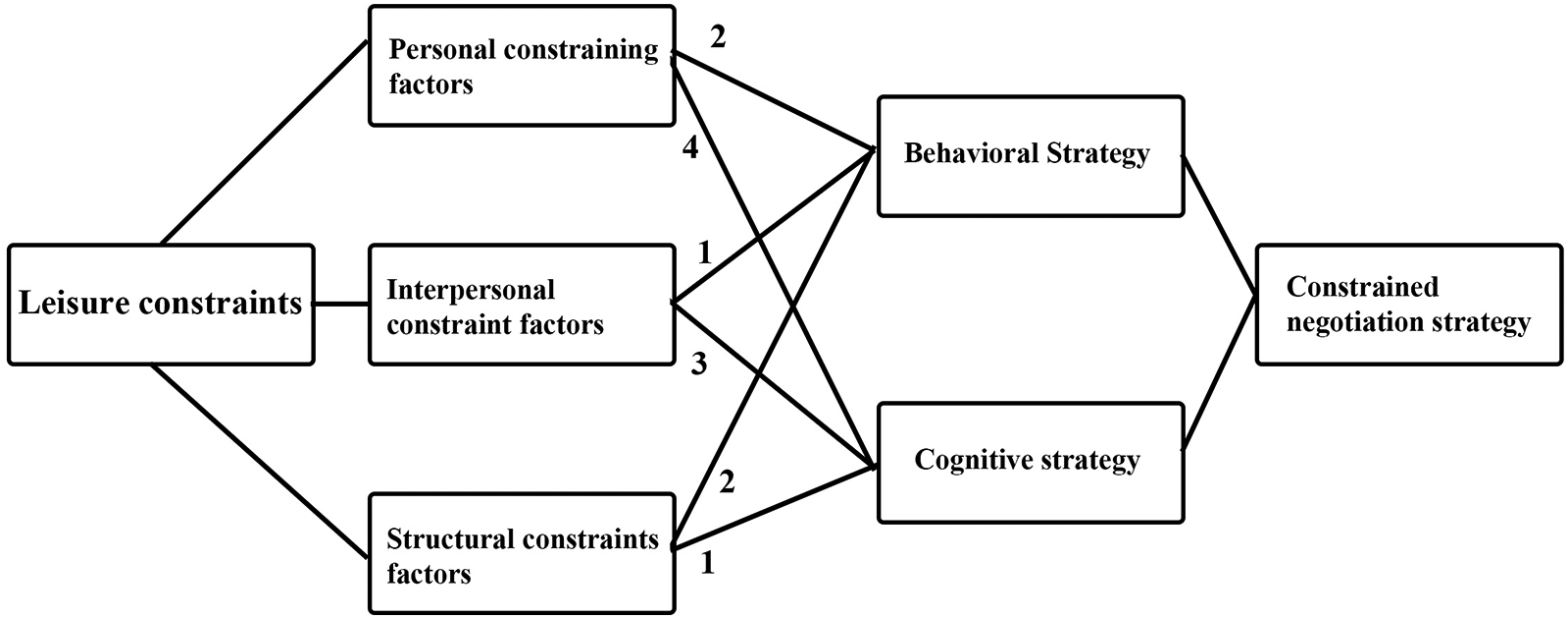
Constraint-consultation structural relationships.

We may observe that there is a specific structural relationship between constraint and negotiation based on the related negotiation methods provided by the interviewees while discussing leisure limitations. While behavioral tactics are primarily employed when addressing structural limits, cognitive strategies are primarily employed when addressing interpersonal and personal constraints. While behavioral tactics were mostly employed to address structural constraints, cognitive strategies were primarily employed to address interpersonal and personal constraints. The particular classification is displayed in Figure 6.

## 4 Conclusions and recommendations

Based on interviews with scuba diving enthusiasts, this study examines the structural relationships between the three dimensions of leisure constraints—intrapersonal constraints (personal), interpersonal constraints (social), and structural constraints (environmental)—and the two strategies of constraint negotiation: cognitive strategies and behavioral strategies. The findings reveal distinct patterns in how enthusiasts address different types of constraints, highlighting the interplay between constraint dimensions and negotiation approaches.

### 4.1 The structural relationships between leisure constraints and constraint negotiation strategies

#### 4.1.1 Intrapersonal and interpersonal constraints (constraints related to “People”)

Constraints related to “people,” such as lack of confidence, fear of safety risks, family opposition, or insufficient social support, are primarily addressed through cognitive strategies. These strategies involve altering psychological perceptions of the activity, modifying expectations, and enhancing self-efficacy. For example, enthusiasts may adjust their mindset by reframing diving as a means of personal growth or stress relief, or by emphasizing the social benefits of engaging with like-minded individuals. By strengthening their psychological and social identification with the role of a diver, enthusiasts develop greater motivation and confidence to continue participating in the activity. This cognitive approach allows them to overcome internal barriers and maintain their enthusiasm for diving.

#### 4.1.2 Structural constraints (constraints related to “Objects”)

Constraints related to “objects”, such as time limitations, financial pressures, or limited access to training opportunities, are predominantly addressed through behavioral strategies. These constraints are more objective in nature and can be tackled through specific actions. For instance, enthusiasts may manage their time by scheduling diving trips around work commitments, seek financial solutions through budgeting or part-time jobs, or join diving clubs to access shared resources and training programs. Behavioral strategies enable enthusiasts to systematically address external barriers, ensuring they can continue to participate in diving despite practical challenges.

#### 4.1.3 Structural relationships between constraints and negotiation strategies

The study identifies a clear structural relationship between the type of constraint and the negotiation strategy employed. Cognitive strategies are predominantly used for intrapersonal and interpersonal constraints, while behavioral strategies are more effective for structural constraints. This relationship underscores the importance of understanding the nature of constraints when designing interventions or support systems for enthusiasts. For example, addressing psychological barriers through community-building initiatives or motivational workshops may be more effective than simply providing financial aid, while improving access to training facilities or flexible scheduling may better address structural limitations. As M1 suggested, “*If there were more affordable training facilities nearby, I would definitely participate more often*”.

### 4.2 Structural relationships: a deeper analysis

The structural relationships between the three dimensions of leisure constraints and the two strategies of constraint negotiation can be further elaborated as follows:

#### 4.2.1 Intrapersonal constraints and cognitive strategies

Intrapersonal constraints, such as lack of confidence or fear of safety risks, are deeply rooted in an individual's psychological state. For instance, regarding lack of confidence, F7 said, “*I was really nervous before my first dive, doubting whether I could handle it. But after completing it successfully, my confidence boosted”*. Cognitive strategies, which involve adjusting perceptions and expectations, are particularly effective in addressing these constraints. By reframing diving as a positive and rewarding experience, enthusiasts can enhance their self-efficacy and reduce anxiety, thereby overcoming personal barriers.

#### 4.2.2 Interpersonal constraints and cognitive strategies

Interpersonal constraints, such as family opposition or lack of social support, often stem from misunderstandings or miscommunications. Cognitive strategies, such as emphasizing the social and recreational benefits of diving, can help enthusiasts gain the support of family and friends. This approach not only addresses the immediate constraint but also strengthens social bonds and identity within the diving community.

#### 4.2.3 Structural constraints and behavioral strategies

Structural constraints, such as time and financial limitations, are external and objective. Behavioral strategies, such as time management and financial planning, are practical and actionable solutions to these challenges. By systematically addressing these external barriers, enthusiasts can continue to participate in diving activities without being hindered by logistical issues.

## 5 Discussion

This study, based on interviews with scuba diving enthusiasts, confirms the structural relationship between leisure constraints and constraint negotiation. The findings align with existing theories, particularly Crawford and Godbey's leisure constraints model and Jackson's classification of constraint negotiation strategies. The study reveals that scuba diving enthusiasts predominantly use cognitive strategies to address personal and interpersonal constraints, while behavioral strategies are more effective for structural constraints. This supports the existing theoretical perspective on the relationship between constraint types and negotiation strategies.

Compared to Western contexts, family dynamics in China play a more pronounced role in shaping interpersonal constraints, with family opposition often stemming from cultural values emphasizing safety and stability. Additionally, structural constraints such as economic pressures and limited access to resources are more pronounced in China, reflecting the country's unique socio-economic landscape. These contextual differences highlight the importance of adapting theoretical frameworks to local cultural and economic conditions.

### 5.1 Contributions of the study

The contributions of this study are significant. First, it fills a research gap in the field of leisure studies in China, where research on scuba diving is relatively scarce. Through empirical research, this study provides new insights into understanding the leisure constraints and negotiation strategies of scuba diving enthusiasts. Second, it enriches theoretical frameworks by validating and refining leisure constraints theory, particularly in terms of the structural relationship between constraint types and negotiation strategies. Third, the findings offer practical implications for diving clubs and related organizations, helping them better understand the constraints faced by scuba diving enthusiasts and design more effective support systems.

### 5.2 Limitations of the study

Despite these contributions, the study has several limitations. The sample size was small, with only 20 scuba diving enthusiasts interviewed, which may limit the generalizability of the results. Additionally, the sample selection was limited, as all respondents were non-professional divers, and most were single and highly educated, which may restrict the diversity of the results. The data collection methods were also limited, relying primarily on interviews without supplementary quantitative data, which may affect the comprehensiveness of the results. Furthermore, self-reported data may be biased, affecting the accuracy and authenticity of the data.

### 5.3 Recommendations for future research

Future research should address these limitations. Expanding the sample size to include a larger and more diverse group of scuba diving enthusiasts would enhance the generalizability and representativeness of the results. Using multiple data collection methods, such as combining surveys and interviews, could provide more comprehensive data. Exploring new analytical methods, such as structural equation modeling (SEM) or machine learning techniques, could deepen the analysis of the relationship between leisure constraints and negotiation strategies. Cross-cultural comparative studies could also explore differences in leisure constraints and negotiation strategies across different cultural contexts. Finally, validating self-reported data through objective measurement methods, such as behavioral observation or physiological measurement, could reduce bias and improve data accuracy.

In summary, this study validates the structural relationship between leisure constraints and negotiation strategies through interviews with scuba diving enthusiasts, providing new empirical support for leisure research. The findings offer valuable references for future research and practice, despite some limitations. Future research should further expand the sample size and methods to more comprehensively reveal the complex relationship between leisure constraints and negotiation strategies.

## Data Availability

The datasets presented in this study can be found in online repositories. The names of the repository/repositories and accession number(s) can be found in the article/Supplementary Material.
