# Correction: Leisure constraints and the negotiation of structural relationships: a case study of scuba diving enthusiasts

**DOI:** 10.3389/fspor.2025.1664741

**Published:** 2025-08-20

**Authors:** Jing Chen, Zihan Yu, Ruiyang Ni

**Affiliations:** ^1^ School of Marxism, Zhejiang Shuren University, Hangzhou, Zhejiang, China; ^2^ Hangzhou International Urbanology Research Center & Zhejiang Urban Governance Studies Center, Hangzhou, Zhejiang, China; ^3^ Zhejiang Leisure Association, Hangzhou, Zhejiang, China; ^4^ School of Public Affairs, Zhejiang Shuren University, Hangzhou, Zhejiang, China; ^5^ School of Humanities & Foreign Languages, Zhejiang Shuren University, Hangzhou, Zhejiang, China

**Keywords:** SCUBA diving, leisure constraints, constraint negotiation, cognitive strategies, behavioral strategies

**Text correction**

In the published article, there was an error in the abstract. Due to multiple submissions, the abstract for another article was mistakenly provided for this article.

The previous abstract was written as:

“**Background and aims:** College students’ social media addiction is linked to psychological anxiety. This study explores the relationship mechanisms, particularly the mediating roles of self-efficacy and coping styles.

**Methods**: Data were collected from 615 college students using questionnaires. Structural equation modeling was employed to analyze the data an investigate both the direct and indirect effects of social media addiction on psychological anxiety through the lens of self-efficacy and coping styles.

**Results:** The results indicated a significant positive correlation between social media addiction and psychological anxiety. Specifically, social media addiction was found to not only directly increase psychological anxiety but also indirectly affect it through two pathways: (1) by reducing self-efficacy, which in turn heightened psychological anxiety, and (2) by influencing coping styles, wherein negative coping styles were positively related to psychological anxiety. Moreover, a chain mediation effect was observed where social media addiction led to decreased self-efficacy, subsequently shifting coping styles and ultimately exacerbating psychological anxiety.

**Discussion and conclusions**: These findings highlight the critical roles of self-efficacy and coping styles in the relationship between social media addiction and psychological anxiety among college students. They provide valuable insights for developing targeted interventions to mitigate the adverse effects of social media addiction on mental health, emphasizing the importance of enhancing self-efficacy and promoting positive coping strategies. By addressing these factors, universities can better support students in maintaining healthy social media habits and reducing psychological anxiety.”

The correct abstract appears below:

“**Background:** Scuba diving has emerged as a popular recreational activity in China over the past two decades, yet academic research on this sport from the perspective of leisure studies remains limited. This study explores the relationship between leisure constraints and constraint negotiation among scuba diving enthusiasts, aiming to fill this research gap.

**Method:** This study employed a mixed-methods approach, combining in- depth interviews with 20 scuba diving enthusiasts and the Positive and Negative Affect Schedule (PANAS) for survey and analysis. The interviews focused on the participants’ leisure motivations, the constraints encountered at different stages of their diving careers, and the negotiation strategies they employed.

**Results:** The findings revealed that scuba diving enthusiasts tend to use cognitive negotiation strategies when addressing personal and interpersonal constraints, while predominantly employing behavioral negotiation strategies when dealing with structural constraints. A structural relationship was identified between leisure constraints and constraint negotiation, indicating that the type of constraint influences the negotiation strategy employed.

**Conclusions**: This study provides empirical support for the structural relationship between leisure constraints and constraint negotiation, enriching the materials available for leisure research. Future research is recommended to expand the sample size and further explore the underlying mechanisms of this relationship, as well as to consider the authenticity and accuracy of respondents’ self-reports.”

**Affiliation correction**

In the published article, there was a translation error in the institution's official English name for affiliation 2.

The affiliation was previously written as:

“Zhejiang Leisure Association, Zhejiang University Zijingang Campus, Hangzhou, Zhejiang, China”

The correct affiliation appears below:

“Zhejiang Leisure Association, Hangzhou, Zhejiang, China”

**Affiliation correction**

In the published article, there was an error in the ordering of affiliations 2 and 3.

The previous affiliations were ordered as:

2. “Zhejiang Leisure Association, Zhejiang University Zijingang Campus, Hangzhou, Zhejiang, China”

3. “Hangzhou International Urbanology Research Center & Zhejiang Urban Governance Studies Center, Hangzhou, Zhejiang, China”

The correct order of affiliations appears below:

2. “Hangzhou International Urbanology Research Center & Zhejiang Urban Governance Studies Center, Hangzhou, Zhejiang, China”

3. “Zhejiang Leisure Association, Hangzhou, Zhejiang, China” The original article has been updated.

